# High occurrence of *BRCA1 *intragenic rearrangements in hereditary breast and ovarian cancer syndrome in the Czech Republic

**DOI:** 10.1186/1471-2350-8-32

**Published:** 2007-06-11

**Authors:** Petra Vasickova, Eva Machackova, Miroslava Lukesova, Jiri Damborsky, Ondrej Horky, Hana Pavlu, Jitka Kuklova, Veronika Kosinova, Marie Navratilova, Lenka Foretova

**Affiliations:** 1 Masaryk Memorial Cancer Institute, Brno, Czech Republic; 2 Loschmidt Laboratories, Faculty of Science, Masaryk University, Brno, Czech Republic; 3 Center of Molecular Biology and Gene Therapy, University Hospital, Brno, Czech Republic

## Abstract

**Background:**

Alterations in the highly penetrant cancer susceptibility gene *BRCA1 *are responsible for the majority of hereditary breast and/or ovarian cancers. However, the number of detected germline mutations has been lower than expected based upon genetic linkage data. Undetected deleterious mutations in the *BRCA1 *gene in some high-risk families could be due to the presence of intragenic rearrangements as deletions, duplications or insertions spanning whole exons. Standard PCR-based screening methods are mainly focused on detecting point mutations and small insertions/deletions, but large rearrangements might escape detection.

The purpose of this study was to determine the type and frequency of large genomic rearrangements in the *BRCA1 *gene in hereditary breast and ovarian cancer cases in the Czech Republic.

**Methods:**

Multiplex ligation-dependent probe amplification (MLPA) was used to examine *BRCA1 *rearrangements in 172 unrelated patients with hereditary breast and/or ovarian cancer syndrome without finding deleterious mutation after complete screening of whole coding regions of *BRCA1/2 *genes. Positive MLPA results were confirmed and located by long-range PCR. The breakpoints of detected rearrangements were characterized by sequencing.

**Results:**

Six different large deletions in the *BRCA1 *gene were identified in 10 out of 172 unrelated high-risk patients: exons 1A/1B and 2 deletion; partial deletion of exon 11 and exon 12; exons 18 and 19 deletion; exon 20 deletion; exons 21 and 22 deletion; and deletion of exons 5 to 14. The breakpoint junctions were localized and further characterized. Destabilization and global unfolding of the mutated BRCT domains explain the molecular and genetic defects associated with the exon 20 in-frame deletion and the exon 21 and 22 in-frame deletion, respectively.

**Conclusion:**

Using MLPA, mutations were detected in 6% of high-risk patients previously designated as *BRCA1/2 *mutation-negative. The breakpoints of five out of six large deletions detected in Czech patients are novel. Screening for large genomic rearrangements in the *BRCA1 *gene in the Czech high-risk patients is highly supported by this study.

## Background

Breast cancer is the most commonly diagnosed cancer in women in Europe today. A hereditary form of breast cancer is characterized by young age onset, increased risk of bilateral breast cancer, and its being frequently in association with ovarian cancer. The existence of an autosomal dominant pattern of inheritance accounting for 5–10% of the breast cancer cases has been demonstrated [1]. Germline mutations in *BRCA1 *(OMIM#113705, Online Mendelian Inheritance in Man) and *BRCA2 *(OMIM#600185) genes are responsible for an important fraction of hereditary breast and ovarian cancers [2]. A few hundred different mutations associated with inherited predisposition to breast and ovarian cancers have been identified in the *BRCA1 *and *BRCA2 *genes, as described at the Breast Cancer Information Core internet web site (BIC database) [3].

Most reported germline deleterious mutations are nonsense substitutions and small deletions/insertions causing truncations of *BRCA1*/*2 *proteins. In most populations tested, the observed frequencies of *BRCA1 *variations in high-risk breast and/or ovarian cancer families have been described as lower than predicted by linkage analysis. Pathogenic mutations in the coding region or in splice sites of the *BRCA1 *gene were found in approximately two-thirds of *BRCA1*-linked families [2]. This finding suggests that methods generally used for mutation scanning fail to detect certain types of *BRCA1 *germline defects, such as large intragenic rearrangements. Most of the screening methods based on the PCR of genomic DNA are qualitative rather than quantitative [4]. Partial or complete exon loss or amplification might be overlooked because of the presence of a wild-type allele that gives rise to a positive PCR signal and therefore a possible false-negative result. Several approaches have been used for detecting *BRCA1 *rearrangements: Southern blot [5-9], long-range PCR [10], color bar coding of the *BRCA1 *gene on combed DNA [11,12], semiquantitative-multiplex PCR [13,14] or real-time PCR [15]. Recently, multiplex ligation-dependent probe amplification (MLPA) has been widely used as a highly sensitive method for detecting the relative copy number of all *BRCA1 *exons in a high-throughput format [16].

Many different *BRCA1 *germline rearrangements with mapped breakpoints have been reported to date [17]. These are scattered throughout the whole gene and most of them are deletions, but duplication, triplication or combined deletion/insertion events also have been described. A genetic structure of *BRCA1 *with extremely high density of intronic *Alu *repeats and the presence of a duplicated promoter region containing a *BRCA1 *pseudogene upstream of the *BRCA1 *could provide hotspots for unequal homologous recombination [18,19]. The proportion of genomic rearrangements in the *BRCA1 *mutation spectrum has been studied in several countries and often was found to vary from 8 to 15% [7,20-23]. Higher values, probably due to a strong founder effect, have been presented by studies performed in the Netherlands and Italy [24,25]. The majority of known rearrangements create frame shifts that result in premature termination of translation. Therefore, the phenotype of patients carrying this type of mutations is not expected to be distinct from patients with other truncating mutations. Most mutations introducing a stop codon into *BRCA1 *have been described as leading to nonsense-mediated decay of mRNA, irrespective of their type [26]. Large deletions of one or more exons maintaining the reading frame could cause loss of putative functional domains of the *BRCA1 *protein [8]. However, no assay for the *BRCA1 *gene is currently available for testing the exact functional consequences of such mutations.

The presence of large rearrangements in the *BRCA1 *gene offers a promising outlook in clinical practice, and especially for probands with previously negative results of *BRCA*1/2 mutation screening. If causative mutation is determined, predictive testing can be performed to identify family members who may benefit from increased surveillance, chemoprevention or prophylactic surgery to reduce the risk of developing cancer [27]. The aim of this study was to determine the frequency and type of *BRCA1 *intragenic rearrangements in Czech high-risk breast and/or ovarian cancer families where no deleterious mutations were previously found and to assess whether testing for such rearrangements should be included in standard mutation screening.

## Methods

### Patients and criteria for testing

The test group was comprised of 172 high-risk Czech families with hereditary breast and/or ovarian cancer syndrome referred for genetic testing to the Masaryk Memorial Cancer Institute in Brno (Table 1). All tested individuals were counseled and gave signed informed consent. The inclusion criteria were as follow: (i) unrelated index patients affected by invasive breast and/or ovarian cancer, (ii) at least three diagnoses of breast and/or ovarian cancer in the family diagnosed at any age (bilateral cancers were counted as two cases), and (iii) no deleterious mutation found during complete screening of the whole coding regions of *BRCA1 *and *BRCA2 *genes as described by Foretova et al. [28]. This group could be divided into two main parts consistent with the cancer phenotype of the probands' families: the individuals belonging to the families with at least one case of ovarian cancer (45 patients) and those individuals from the families with breast cancer phenotype only (127 patients). These were further subdivided according to the number of individuals diagnosed with breast cancer at ages under 50 years (Table 1).

**Table 1:** Molecular genetic testing in 290 Czech high-risk families

Phenotype of families tested for *BRCA1/2 *mutations*	Number of families/patients	*BRCA1 *mutation (%)	*BRCA2 *mutation (%)	Overall mutation (%)	Phenotype and number of families tested for *BRCA1 *deletions*	**Number of *BRCA1 *deletions detected**
HBOC+HOC	105	51 (48.6%)	9 (8.6%)	60 (57.2%)	HBOC **36**	**4**
					HOC **9**	**1**
					Σ **45**	**5/45 (11.1%)**
					5/56 **~8.93% of *BRCA1 *mutations**	
HBC	185	35 (18.9%)	23 (12.4%)	58 (31.3%)	**0 **× brca<50 **29**	**-**
					**1 **× brca<50 **52**	**1**
					**2 **× brca<50 **28**	**1**
					**3 **× brca<50 **15**	**-**
					**4 **× brca<50 **3**	**3**
					Σ **127**	**5/127 (3.9%)**
					5/40 **~12.5% of *BRCA1 *mutations**	
					**Overall 172**	**10/172 (5.8%)**
					10/96 **~10.4% of *BRCA1 *mutations**	

Overall	**290**	**86 (29.7%)**	**32 (11.0%)**	**118 (40.7%)**		**10/290 (3.4%)**

*See Materials and methods, Patients and criteria for testing. Only deleterious mutations are considered. HBOC – hereditary breast and ovarian cancer syndrome; HOC – hereditary ovarian cancer syndrome; HBC – hereditary breast cancer syndrome.

### Detecting large genomic rearrangements using MLPA

Relative quantification of the copy numbers of all 24 *BRCA1 *exons was performed by the Salsa P002 *BRCA1 *MLPA probe mix assay (M.R.C. Holland, Amsterdam, the Netherlands) as described by the manufacturer [29]. Each MLPA analysis was carried out on 10 samples and 2 controls on a PTC-200 thermal cycler (Bio-Rad, Hercules, CA, USA). PCR products were separated on an ALFexpress™ II (Amersham Pharmacia Biotech, Uppsala, Sweden) or an ABI PRISM 310 (Applied Biosystems, Foster City, USA) instrument. A peak pattern of 34 peaks ranging in size from 127 to 454 bp was detected [16]. The data obtained on the capillary sequencer ABI-310 were analyzed using GeneScan 3.1.2 Software. The peak heights were normalized and deletions were suspected when the peak height was lower than 65% of the controls. The positive MLPA results were confirmed using new DNA samples in independent assays. DNA sequence analysis of the appropriate ligation sites was done in the case of single exon deletions to eliminate the possibility of an amplification artifact or a presence of polymorphism in ligation sites. False-positive deletions or duplications of single exons were resolved by repeated testing of independent DNA samples for the patient.

### Confirmation and characterization of the rearrangements

Positive results detected by MLPA of two independently drawn samples of genomic DNA were confirmed by long-range PCR (Expand Long Template PCR System, Roche Applied Science), conducted in accordance with the manufacturer's instructions. Several pairs of primers located in exons or introns flanking the rearrangements were used to localize the breakpoint junctions. The GeneFisher program was used for primer design [30]. Selected primers used for long-range PCR of detected Czech rearrangements are presented in Table 2. PCR products were separated by agarose gel electrophoresis and visualized by ethidium bromide staining. A smaller fragment corresponding to the allele with deletion compared to the wild-type allele was obtained in all six deletions detected in this study. Such aberrant DNA fragments were cut out and isolated from agarose gel (QIAquick gel extraction Kit, QIAGEN, Hilden, Germany), sequenced with appropriate primers (ThermoSequenase Cy5 Dye Terminator Cycle Sequencing Kit, Amersham Biosciences, UK), and analyzed on an ALFexpress™ II sequencer (Amersham Pharmacia Biotech, Uppsala, Sweden) or an ABI 3130 genetic analyzer (Applied Biosystems, Foster City, USA). The Repeat Masker program was used to identify *Alu *sequences at breakpoint junctions [31].

**Table 2:** PCR primers used for long-range PCR

**Affected exons – primer pair**	GenBank: L78833^a^	**Size^**b **^[kb]**	**Sequence 5' > 3'**
**1A/1B-2**	Puget et al. [19]	~5	TCAAGGAAATTTTCTTTTGTGC [19] TGTGGAGTTTCCCCCATTCT [19]
**5–14**	19244–54463	3.6	CCTTACCTACCTACATTCAC CTTTATGTAGGATTCAGAGTA
**11–12**	34650–41932	0.72	AGGAGCATTTGTTACTGAG AGAGAGAAAAGGCCTCCTA
**18–19**	63463–66158	0.76	CACAGGGTCAGAGGGTAG AGAGGATATCCTGGTTTGC
**20**	67298–76514	2.2	AGTCCCTGGTAGGATTCAG TATTGAGCACTGGAGATGTG
**21–22**	76247–81170	1.4	TGCCACCAGCCACATG AGCACCAGGTAATGAGTGATAA

^a^Region of *BRCA1 *genomic sequence amplified by primer pair (nucleotide position, [GenBank: L78833]).

^b^Deleted allele.

### Nomenclature

Detected mutations were described at the gDNA level according to the GenBank Database *BRCA1 *reference sequence L78833 (or to the sequence of BAC clone AC060780 in case of the deletion of 1A/1B-2 exons) and following the recommended nomenclature system for human gene mutations [32].

### Structural interpretation of mutations

The crystal structures of the BRCT domains of the *BRCA1 *protein [33] (PDB-ID 1JNX) and its complexes with the tumor suppressor p53 [34] (PDB-ID 1KZY), with the phosphorylated bach1 peptide [35] (PDB-ID 1T29), with the phosphopeptides [36,37] (PDB-IDs 1T2V and 1Y98), and with the phosphorylated interacting region from bach1 helicase [38] (PDB-ID 1T15) were inspected using the PyMol viewer v0.99 (DeLano Scientific, LLC).

## Results and discussion

**Figure 1:**
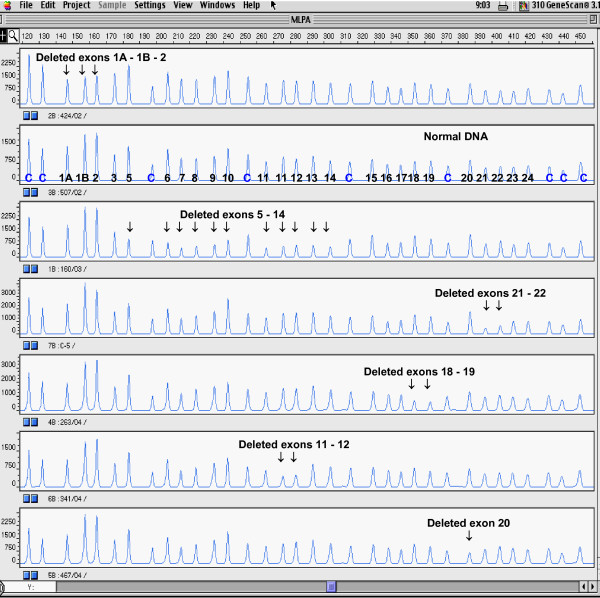
**Multiplex ligation-dependent probe amplification (MLPA) electropherogram (ABI PRISM 310 genetic analyzer, Applied Biosystems)**. Probe mix P002 contains 34 probes: nine control probes recognizing non-*BRCA1 *sequences on various chromosomes are indicated by **"c"**; exons recognized by the *BRCA1*-specific probes are indicated by numbers (probes for both alternative exons 1...1A, 1B; exon 4 is not present in normal *BRCA1 *transcript; two probes specific for exon 11 are included). Note decreased peak heights of deleted exons.

The MLPA analysis of genomic DNA of 172 affected individuals from high-risk families with hereditary breast and/or ovarian cancer syndrome revealed six different deletions covering 1.9–36.9 kb of the *BRCA1 *genomic DNA (Figure 1, Table 3). The average age at the onset of the cancer diagnosis in probands with detected *BRCA1 *rearrangements was 40 years. The effect of decreasing age at onset of the illness in younger generations could be observed in some families. Except for two rearrangements including the exons 5–14 and 11–12, the deletions spanning the exons 1A/1B-2, 18–19, 20 and 21–22 had been previously described in the literature [6,8,19,25,39]. However, the breakpoints of five out of six deletions detected in Czech patients were different from those characterized previously (Table 3).

**Table 3:** *BRCA1 *germline rearrangements identified in the *BRCA1 *gene

***BRCA1 *exons involved**	**Confirmation by long-range PCR**	**Mutation designation **[GenBank: L78833]	**Suspected minimal effect on mRNA**^**a**^	**Fenotype**^**b**^	**Number of families identified**	**Sequence at breakpoint 5'/3'**
**1–2**	yes	Preisler-Adams et al. del 36.9 kb^c ^[40]	not expressed [19]	HBOC	1	ψ gene/gene HR [40]
**5–14**	yes	g.21716_53298 del31583	loss of 3/4 of coding sequence	HBC HBOC	4	LINE1/-
**Part of 11–12**	yes	g.34845_41405 del6561	loss of 1/2 of coding sequence	HOC	1	-/LINE1
**18–19**	yes	g.63651_65590 del1940	p.Asp1692Ala fsX2	HBOC	1	*Alu*Y/*Alu*Sp
**20**	yes	g.68764_75792 del7029	p.His1732_Lys1759del	HBC	1	*Alu*Sq/*Alu*Sx
**21–22**	yes	g.77128_80906 del3779ins236	p.Ile1760_Thr1802del	HBC	2	*Alu*Sx/*Alu*Jb

^a^Inferred from change at the DNA level.

^b^HBC – human breast cancer, HBOC – human breast and ovarian cancer, HOC – human ovarian cancer.

^c^Based on the BAC clone sequence AC060780.

**Figure 2:**
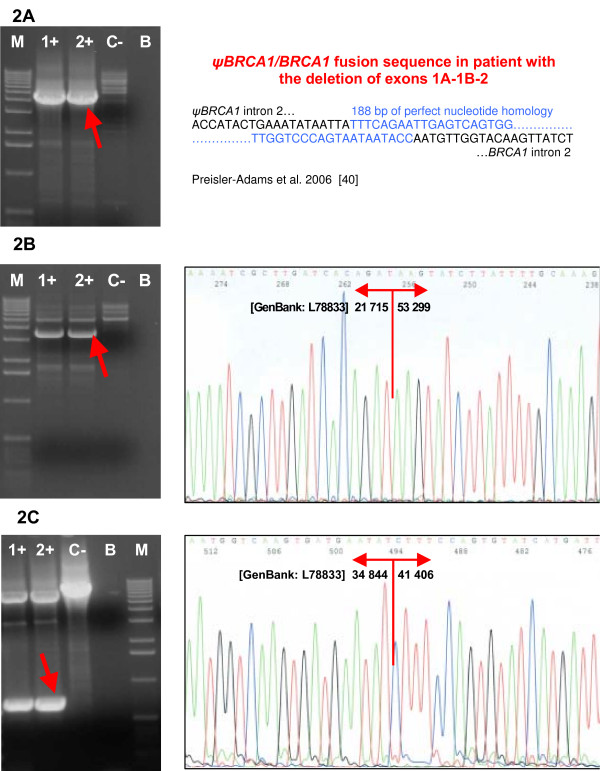
**Confirmation and characterization of the rearrangements**. **(A) **Confirmation of the deletion of exons 1A/1B-2 by long-range PCR and sequencing of the breakpoints. **(B) **Confirmation of the deletion of exons 5–14 by long-range PCR and sequencing of the breakpoints. **(C) **Confirmation of the deletion of exons 11–12 by long-range PCR and sequencing of the breakpoints. Lanes 1+, 2+, carriers of the deletion; lane C-, negative control (wt); lane B, blank; lane M, marker (Ready-Load™ 1 Kb DNA Ladder, Invitrogen).

The deletion of 1A/1B-2 exons was detected by MLPA in one family with the mother affected with ovarian cancer at age 43 and with two relatives affected with breast cancer at young age: her daughter at the age of 38 and her sister at age 39. The deletion of 1A/1B-2 exons was confirmed by long-range PCR with primers published by Puget et al. [19] (Figure 2A). The intensive PCR product was created only for the mutant allele and sequencing revealed the 36.9 kb deletion extending from intron 2 of the *ψBRCA1 *to intron 2 of the *BRCA1 *gene. The breakpoint region is consistent with that reported previously by Preisler-Adams et al. [40] and comprises 188 bp of perfect nucleotide homology between sequences located in the intron 2 of *ψBRCA1 *and intron 2 of the *BRCA1 *gene (Figure 2A). Based on the BAC clone sequence AC060780, the breakpoints occurred between nucleotides 71053–71240 in *ψBRCA1 *and nucleotides 34118–34305 in *BRCA1 *intron 2 [40]. The mutant allele harbors a chimeric gene consisting of the *ψBRCA1 *exons 1A, 1B and 2 fused to the *BRCA1 *exons 3–24 as a result of recombination between sequences located in the intron 2 of *ψBRCA1 *and intron 2 of the *BRCA1 *gene. The promoter was shown to be absent from this mutant allele and expression of the mutant allele was not observed [19,25].

Deletion of the *BRCA1 *exons 5 to 14 was revealed by MLPA in four high-risk, severely affected families with young-age onset of cancer: two families with the hereditary breast-ovarian cancer syndrome and two families with only the breast cancer syndrome. Long-range PCR confirmed a deletion of 31.5 kb of genomic DNA spanning more than three-quarters of the *BRCA1 *gene coding sequence. This deletion interferes with the RING domain (N-terminal zinc finger domain) of the *BRCA1 *protein as well as with important interaction domains for multiple proteins, and it is suggestive of having a negative impact on the function of the *BRCA1 *protein. The exact breakpoints were characterized by sequencing as g.21716_53298del31583 (Figure 2B, Table 3). The breakpoint junctions determined in all four families were identical, thus supporting the likelihood of the founder effect. Moreover, three of the four individuals with confirmed deletion of exons 5–14 came from the same geographical region of the Czech Republic. Only haplotype analysis could reveal if repeated observation of these four deletions is due to the presence of founder mutation or only to the local instability in a region [7,22].

**Figure 3:**
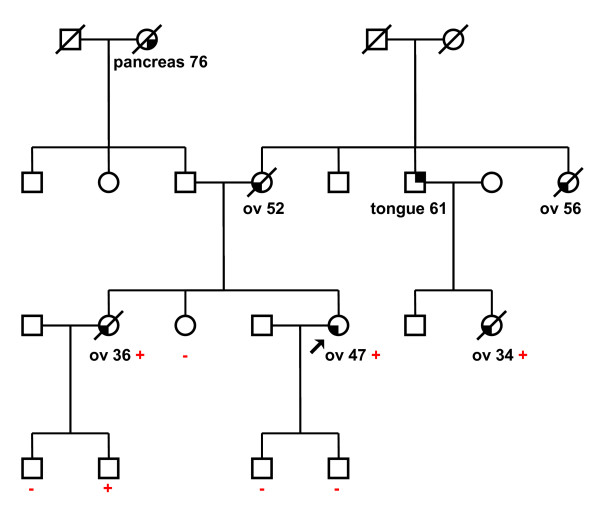
**Pedigrees of ovarian cancer family with detected deletion of *BRCA1 *exons 11–12 (g.34845_41405del6561)**. Circles – females, squares – males, partially filled symbols – affected individuals, arrow – proband. Type of cancer and age of onset are indicated below each affected individual (ov – ovarian). Mutation status: + carrier, - no carrier (wt).

The deletion of the second part of the exon 11 and exon 12 was found by MLPA in a family with ovarian cancer phenotype only. Altogether, five women in two generations were affected with ovarian cancer at ages ranging from 34 to 56 years (Figure 3). Long-range PCR confirmed a deletion of 6.5 kb of genomic DNA spanning nearly one-half of the *BRCA1 *gene coding sequence. The exact breakpoints were characterized by sequencing as g.34845_41405del6561 (Figure 2C, Table 3). An aberrant splicing of mRNA that might further extend a defect on the *BRCA1 *protein is suspected in this case. This deletion lies within a central-risk region where mutations were associated with a significantly higher ovarian/breast cancer ratio [41].

**Figure 4:**
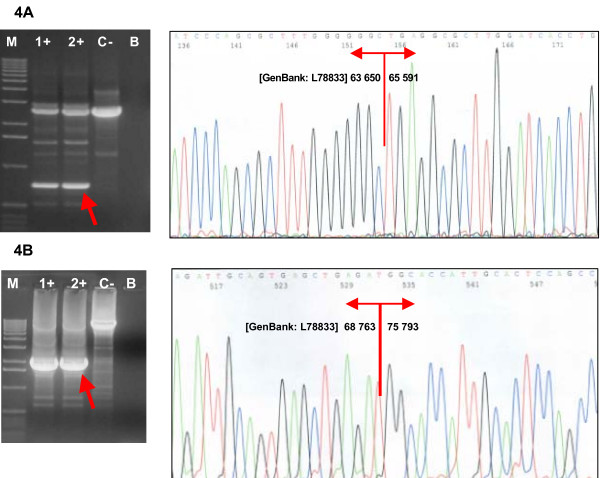
**Confirmation and characterization of the rearrangements**. **(A) **Confirmation of the deletion of exons 18–19 by long-range PCR and sequencing of the breakpoints. **(B) **Confirmation of the deletion of exon 20 and sequencing of the breakpoints.Lanes 1+, 2+, carriers of the deletion; lane C-, negative control (wt); lane B, blank; lane M, marker (Ready-Load™ 1 Kb DNA Ladder, Invitrogen).

The deletion of the exons 18 and 19 was revealed by MLPA in one woman diagnosed with both breast and ovarian cancers at the ages of 52 and 59, respectively, and with a family history of breast cancer in her second-degree relatives. Long-range PCR confirmed a deletion of nearly 2 kb of *BRCA1 *genomic DNA. The exact breakpoints were characterized by sequencing as g.63651_65590del1940 (Figure 4A, Table 3). The out-of-frame deletion of exons 18–19 is predicted to result in a truncation of the *BRCA1 *protein at the codon 1693. As displayed previously, most truncating mutations of the *BRCA1 *gene lead to nonsense-mediated mRNA decay and an allelic imbalance in the expression of the mutant versus wild-type allele [26,42].

The deletion of the exon 20 was found by MLPA in a woman affected with breast and colorectal cancers at the ages of 37 and 35, respectively. Her mother was affected with bilateral breast cancer at the ages of 39 and 46. Long-range PCR confirmed a deletion of about 7 kb of *BRCA1 *genomic DNA. The exact breakpoints were characterized by sequencing as g.68764_75792del7029 (Figure 4B, Table 3). The genomic deletion of exon 20 causes at least in-frame deletion of exon 20 in mRNA and results in the removing of 28 amino acids of the *BRCA1 *protein in position 1732–1759. This deletion affects the highly conserved area of the *BRCA1 *protein corresponding to the linker between two BRCT (*BRCA1 *C-terminal) domains, and it has been described to be involved in DNA repair and transcription activation [43].

**Figure 5:**
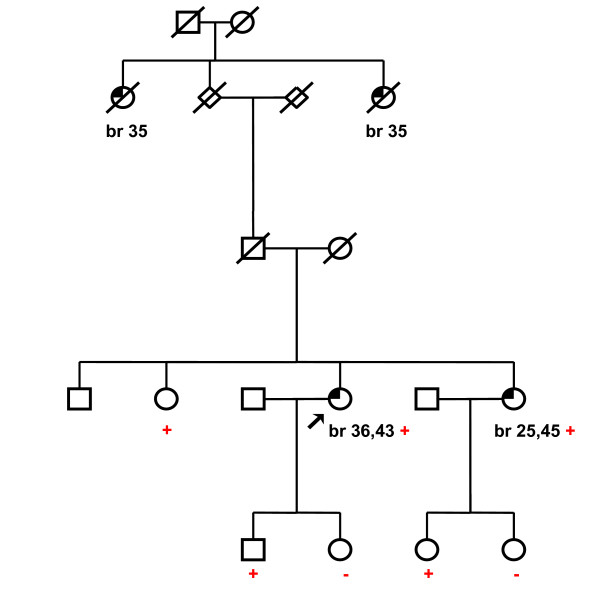
**Pedigrees of breast cancer family with detected deletion of *BRCA1 *exons 21–22 (g.77128_80906del3779ins236)**. Circles – females, squares – males, partially filled symbols – affected individuals, arrow – proband. Type of cancer and age of onset are indicated below each affected individual (br – breast). Mutation status: + carrier, - no carrier (wt).

**Figure 6:**
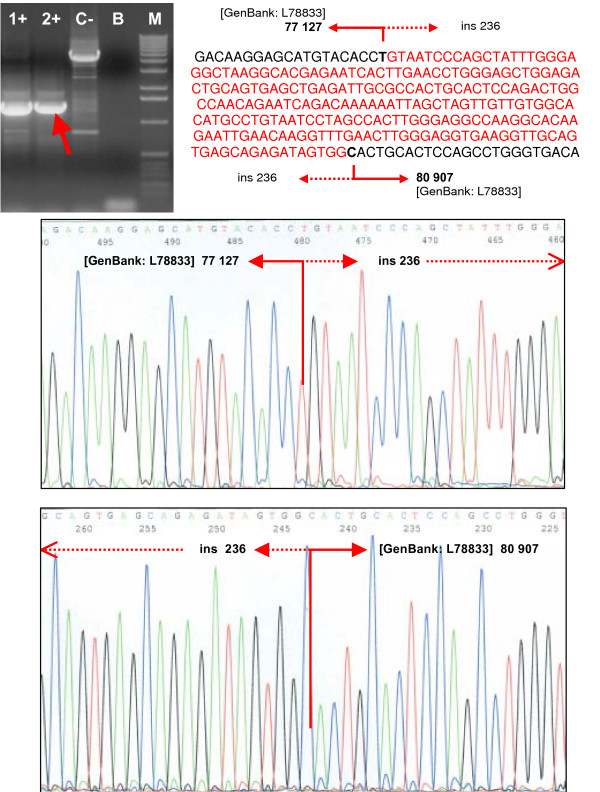
**Confirmation and characterization of the rearrangements**. Confirmation of the deletion of the exons 21–22 by long-range PCR and sequencing of the breakpoints. The deletion/insertion event was characterized as g.77128_80906del3779ins236. Lanes 1+, 2+, carriers of the deletion; lane C-, negative control (wt); lane B, blank; lane M, marker (Ready-Load™ 1 Kb DNA Ladder, Invitrogen).

The next in-frame deletion of the exons 21 and 22 was detected by MLPA in two high-risk families with hereditary breast cancer phenotype only (Figure 5). Long-range PCR confirmed a deletion of about 3.5 kb of *BRCA1 *genomic DNA. Sequencing of the breakpoint's region revealed the deletion/insertion event characterized as g.77128_80906del3779ins236 (Figure 6, Table 3). However, the in-frame deletion of exons 21–22 removes at least 43 amino acids of the *BRCA1 *protein in position 1760–1802, corresponding to a part of the C-terminal BRCT domain.

**Figure 7:**
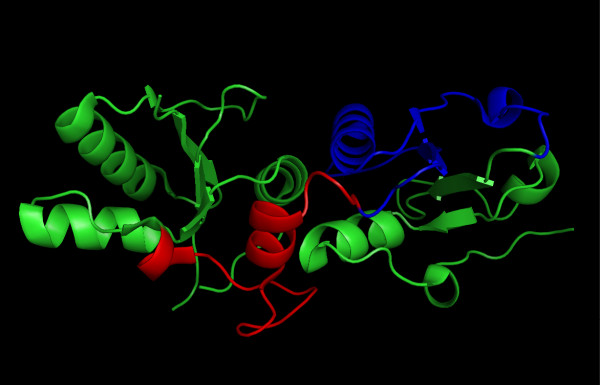
**A ribbons representation of the BRCT domain**. The deletion of 28 amino acids encoded by the exon 20 correspond to the linker (colored red). The deletion of 43 amino acids encoded by the exons 21 and 22 correspond to the C-terminal repeat of BRCT (colored blue). See Results.

The loss of a part of the conservative domain of *BRCA1 *protein might have an effect on protein function and is suspected to be causative of cancer susceptibility. Even missense mutations located in this region (for example, P1749R and M1775R) were described to segregate with the disease and to have a destabilizing effect on the BRCT domain [44]. However, the exact changes in the function of the *BRCA1 *protein cannot be determined because a functional assay is lacking. Therefore, the structural model of deletions in the BRCT domain was constructed to help with the interpretation of an effect on the stability of the BRCT domain (Figure 7), which might be indirectly related to the disease risk [44].

Inspection of the crystal structures of the BRCT repeat region from the *BRCA1 *revealed that the mutant with genomic deletion of exon 20 encodes the protein with the missing linker region between the N-terminal and the C-terminal BRCT repeats of *BRCA1*, while the in-frame deletion of exons 21 and 22 results in the protein with the missing C-terminal repeat of BRCT (Figure 7). The two BRCT domains interact in a head-to-tail fashion, burying about 1600 A^2 ^of solvent-accessible surface area in the interface [45]. The truncation of the linker or the exclusion of any of the two BRCT domains will result in the exposure of the interface and the protein's unfolding (Figure 7). It is obvious that the protein with a disrupted carboxyl-terminal BRCT repeat region cannot fulfill its tumor suppressor function. This structural interpretation is in accordance with the study of Williams et al. [44,46], who used a protease-based assay to assess the sensitivity of the folding of the BRCT domain to an extensive set of truncation and single amino acid substitutions derived from breast cancer screening programs. The protein can tolerate truncations of up to eight amino acids, but further deletion leads to BRCT folding defects.

It would have been interesting to determine whether detected deletions segregate with disease in affected families, but no informative data are available. In families with the deletion of exons 11+12 and the deletion of exons 21+22, all affected patients tested (3 and 2) were found to be carriers of the deletion (Figs. 3 and 5). In the cases of the remaining eight families, as there were no other affected individuals alive, only healthy individuals from mainly younger generations could be tested.

Most of the previously characterized rearrangements in the *BRCA1 *gene result from an unequal homologous recombination of *Alu *repeats [17]. The presence of *Alu *elements revealed by the Repeat Masker program in breakpoint junctions in three out of five novel deletions characterized in this study supports this hypothesis. Contrary to the *Alu*-mediated deletions of exons 18–19, 20 and 20–21, no *Alu *repeats were found in the breakpoints of the deletions including exons 11–12 and 5–14 (Table 3). In these two cases, L1 repetitive sequences present near one side of the breakpoint do not correlate with any recognizable repeat motifs opposite, suggesting nonhomologous events or other mechanisms [10,39]. The role of the L1 repetitive elements in relation to the *BRCA1 *intragenic rearrangements has not yet been mentioned.

The intragenic deletions were detected in almost 6% of all high-risk families previously considered negative for the mutation in *BRCA1 *or *BRCA2 *genes. It represents 10.4% of all detected *BRCA1 *mutations and 7.8% of all mutations detected in *BRCA1 *and *BRCA2 *genes (in 290 high-risk families tested for mutations during 1999–2006, Table 1). This ratio is dependent on the selection of patients and the mutation detection rate. As can be seen from Table 1, *BRCA1 *rearrangements were found either in breast plus ovarian cancer families (5 cases) or in breast cancer families with at least one individual with breast cancer diagnosed under 50 years of age (5 cases). The deletions were identified in all three families, each with four individuals affected by breast cancer under age 50. By contrast, no rearrangement was detected in families with late-onset multiple breast cancer cases. In affected families, ovarian cancer or at least one case of breast cancer under age 50 seems to suggest the presence of *BRCA1 *gross rearrangement.

The proportion of intragenic *BRCA1 *rearrangements could be overestimated because of a higher objective amount of pathogenic mutations in the Czech population. *BRCA1/2 *mutations were excluded in this study using a combination of heteroduplex analysis and protein truncation tests. Heteroduplex analysis is aimed to detect small insertions and deletions and not at detecting single base changes. The protein truncation test is a convenient method for rapidly scanning relatively large fragments for protein-terminating variants, but it is incapable of identifying some potentially risky missense variants or small in-frame deletions located in exon 11 of *BRCA1 *or exon 10 and 11 of *BRCA2*.

Our results are in good concordance with those obtained by studies performed in other countries: the proportion of the *BRCA1 *intragenic rearrangements is slightly higher than are those estimated in France, Germany, Spain and in the United States [7,20,21,23] but lower than in the populations of Australia or New Zealand [22]. Higher proportions of *BRCA1 *rearrangements have been observed in the Netherlands, due to the founder mutations representing 23% of all *BRCA1 *mutations found [24], and in a small population in Northern Italy [25]. On the other hand, a study performed in Finland failed to detect any rearrangements in the *BRCA1 *gene [47]. The most likely explanation for varying prevalencies of large rearrangements is in the differing genetic backgrounds of the populations studied. The study size and selection bias may be relevant, too. Methodology is not supposed to be a major factor, because the same commercially available MLPA kit is widely used. Our results indicate that MLPA is a rapid, reliable and sensitive technique allowing high-throughput screening for the *BRCA1 *rearrangements.

## Conclusion

Using MLPA technique, intragenic rearrangements were detected in approximately 6% of the Czech high-risk families previously designated as *BRCA1/2 *mutation negative. Six different intragenic deletions represent more than 10% of all detected *BRCA1 *mutations. Our results prove the usefulness of testing for large *BRCA1 *rearrangements in the Czech population. These results are important for counseling purposes and clinical management of patients as well as for the possibility of predictive testing of relatives. MLPA testing of the *BRCA2 *rearrangements is now under examination and might further improve the sensitivity of testing.

## Competing interests

The author(s) declare that they have no competing interests.

## Authors' contributions

PV and EM contributed equally to this work.

PV, EM and LF participated in design of this study.

PV, EM and ML selected patients and their family members appropriate for this study, carried out molecular analyses and interpreted the results.

LF and MN participated in genetic counseling and selection of patients.

PV drafted the paper and finalized the manuscript with help of EM and LF.

OH carried out capillary electrophoresis on an ABI PRISM 310 genetic analyzer.

JD performed structural analysis of BRCT domain and its interpretation.

HP, VK and JK participated in mutation screening.

All authors read and approved the final manuscript.

## Pre-publication history

The pre-publication history for this paper can be accessed here:



## References

[B1] Newman B, Austin MA, Lee M, King MC (1988). Inheritance of human breast cancer: evidence for autosomal dominant transmission in high-risk families. Proc Natl Acad Sci USA.

[B2] Ford D, Easton DF, Stratton M, Narod S, Goldgar D, Devilee P, Bishop DT, Weber B, Lenoir G, Chang-Claude J, Sobol H, Sobol H, Teare MD, Strueving J, Arason A, Scherneck S, Peto J, Rebbeck TR, Tonin P, Neuhausen S, Barkardottir R, Eyfjor J, Lynch H, Ponder BAJ, Gayther SA, Birch JM, Lindblom A, Stoppa-Lyonet D, Bignon Y, Borg A, Hamann U, Haites N, Scott RJ, Maugard CM, Vasen H, Seitz S, Cannon-Albright LA, Schofield A, Zelada-Hedman M, the Breast Cancer Linkage Consortium (1998). Genetic heterogeneity and penetrance of the *BRCA1 *and *BRCA2 *genes in breast cancer families. Am J Hum Genet.

[B3] Breast Cancer Information Core internet web site. http://www.nhgri.nih.gov/Intramural_research/Lab_transfer/Bic/index.html.

[B4] Armour JAL, Barton DE, Cockburn DJ, Taylor GR (2002). The detection of large deletions or duplications in genomic DNA. Hum Mutat.

[B5] Petrij-Bosch A, Peelen T, van Vliet M, van Eijk R, Olmer R, Drusedau M, Hogervorst FB, Hageman S, Arts PJ, Ligtenberg MJ, Meijers-Heijboer H, Klijn JG, Vasen HF, Cornelisse CJ, van 't Veer LJ, Bakker E, van Ommen GJ, Devilee P (1997). *BRCA1 *genomic deletions are major founder mutations in Dutch breast cancer patients. Nat Genet.

[B6] Swensen J, Hoffman M, Skolnick MH, Neuhausen SL (1997). Identification of a 14 kb deletion involving the promoter region of *BRCA1 *in a breast cancer family. Hum Mol Genet.

[B7] Puget N, Stoppa-Lyonet D, Sinilnikova OM, Pages S, Lynch HT, Lenoir GM, Mazoyer S (1999). Screening for germ-line rearrangements and regulatory mutations in *BRCA1 *led to the identification of four new deletions. Cancer Res.

[B8] Rohlfs EM, Chung CH, Yang Q, Skrzynia C, Grody WW, Graham ML, Silverman LM (2000). In-frame deletions of *BRCA1 *may define critical functional domains. Hum Genet.

[B9] Unger MA, Nathanson KL, Calzone K, Antin-Ozerkis D, Shih HA, Martin AM, Lenoir GM, Mazoyer S, Weber BL (2000). Screening for genomic rearrangements in families with breast and ovarian cancer identifies *BRCA1 *mutations previously missed by conformation-sensitive gel electrophoresis or sequencing. Am J Hum Genet.

[B10] Payne SR, Newman B, King MC (2000). Complex germline rearrangements of *BRCA1 *associated with breast and ovarian cancer. Genes Chromosomes Cancer.

[B11] Gad S, Aurias A, Puget N, Mairal A, Schurra C, Montagna M, Pages S, Caux V, Mazoyer S, Bensimon A, Stoppa-Lyonnet D (2001). Color bar coding the *BRCA1 *gene on combed DNA: a useful strategy for detecting large gene rearrangements. Genes Chromosomes Cancer.

[B12] Gad S, Klinger M, Caux-Moncoutier V, Pages-Berhouet S, Gauthier-Villars M, Coupier I, Bensimon A, Aurias A, Stoppa-Lyonnet D (2002). Bar code screening on combed DNA for large rearrangements of the *BRCA1 *and *BRCA2 *genes in French breast cancer families. J Med Genet.

[B13] Casilli F, Di Rocco ZC, Gad S, Tournier I, Stoppa-Lyonnet D, Frebourg T, Tosi M (2002). Rapid detection of novel *BRCA1 *rearrangements in high-risk breast-ovarian cancer families using multiplex PCR of short fluorescent fragments. Hum Mutat.

[B14] Hofmann W, Görgens  H, John A, Horn D, Hüttner  Ch, Arnold N, Scherneck S, Schackert HK (2003). Screening for large rearrangements of the *BRCA1 *gene in German breast or ovarian cancer families using semi-quantitative multiplex PCR method. Hum Mutat.

[B15] Barrois M, Bieche I, Mazoyer S, Champeme MH, Bressac de-Paillerets B, Lidereau R (2004). Real-time PCR-based gene dosage assay for detecting *BRCA1 *rearrangements in breast-ovarian cancer families. Clin Genet.

[B16] Schouten JP, McElgunn CJ, Waaijer R, Zwijnenburg D, Diepvens F, Pals G (2002). Relative quantification of 40 nucleic acid sequences by multiplex ligation-dependent probe amplification. Nucleic Acids Res.

[B17] Mazoyer S (2005). Genomic rearrangements in the *BRCA1 *and *BRCA2 *genes. Hum Mutat.

[B18] Smith TM, Lee MK, Szabo CI, Jerome N, McEuen M, Taylor M, Hood L, King MC (1996). Complete genomic sequence and analysis of 117 kb of human DNA containing the gene *BRCA1*. Genome Res.

[B19] Puget N, Gad S, Perrin-Vidoz L, Sinilnikova OM, Stoppa-Lyonnet D, Lenoir GM, Mazoyer S (2002). Distinct *BRCA1 *rearrangements involving the *BRCA1 *pseudogene suggest the existence of a recombination hot spot. Am J Hum Genet.

[B20] Gad S, Caux-Moncoutier V, Pages-Berhouet S, Gauthier-Villars M, Coupier I, Pujol P, Frenay M, Gilbert B, Maugard C, Bignon YJ, Chevrier A, Rossi A, Fricker JP, Nguyen TD, Demange L, Aurias A, Bensimon A, Stoppa-Lyonnet D (2002). Significant contribution of large genomic rearrangements in 120 French breast cancer families. Oncogene.

[B21] Hartmann C, John AL, Klaes R, Hofmann W, Bielen R, Koehler R, Janssen B, Bartram CR, Arnold N, Zschocke J (2004). Large *BRCA1 *gene deletions are found in 3% of German high-risk breast cancer families. Hum Mutat.

[B22] Woodward AM, Davis TA, Silva AG, Kirk JA, Leary JA, kConFab Investigators (2005). Large genomic rearrangements of both *BRCA2 *and *BRCA1 *are a feature of the inherited breast/ovarian cancer phenotype in selected families. J Med Genet.

[B23] de la Hoya M, Gutierrez-Enriquez S, Velasco E, Osorio A, de Abajo AS, Vega A, Salazar R, Esteban E, Llort G, Gonzalez-Sarmiento R, Carracedo A, Benitez J, Miner C, Diez O, Diaz-Rubio E, Caldes T (2006). Genomic rearrangements at the *BRCA1 *locus in Spanish families with breast/ovarian cancer. Clinical Chemistry.

[B24] Hogervorst FB, Nederlof PM, Gille JJ, McElgunn CJ, Grippeling M, Pruntel R, Regnerus R, van Welsem T, van Spaendonk R, Menko FH, Kluijt I, Dommering C, Verhoef S, Schouten JP, van't Veer LJ, Pals G (2003). Large genomic deletions and duplications in the *BRCA1 *gene identified by a novel quantitative Method. Cancer Res.

[B25] Montagna M, Dalla Palma M, Menin C, Agata S, De Nicolo A, Chieco-Bianchi L, D'Andrea E (2003). Genomic rearrangements account for more than one-third of the *BRCA1 *mutations in northern Italian breast/ovarian cancer families. Hum Mol Genet.

[B26] Perrin-Vidoz L, Sinilnikova OM, Stoppa-Lyonnet D, Lenoir GM, Mazoyer S (2002). The nonsense-mediated mRNA decay pathway triggers degradation of most *BRCA1 *mRNAs bearing premature termination codons. Hum Mol Genet.

[B27] Wooster R, Weber BL (2003). Breast and ovarian cancer. N Engl J Med.

[B28] Foretova L, Machackova E, Navratilova M, Pavlu H, Hruba M, Lukesova M, Valik D (2004). *BRCA1 *and *BRCA2 *mutations in women with familial or early-onset breast/ovarian cancer in the Czech Republic. Hum Mutat.

[B29] MRC-Holland. http://www.mrc-holland.com.

[B30] GeneFisher – Interactive PCR Primer Design. http://bibiserv.techfak.uni-bielefeld.de/genefisher/.

[B31] RepeatMasker Web Server. http://www.repeatmasker.org/cgi-bin/WEBRepeatMasker.

[B32] Antonarakis SE, the Nomenclature Working Group (1998). Recommendations for a nomenclature system for human gene mutations. Hum Mutat.

[B33] Williams RS, Green R, Glover JN (2001). Crystal structure of the BRCT repeat region from the breast cancer-associated protein *BRCA1*. Nat Struct Biol.

[B34] Joo WS, Jeffrey PD, Cantor SB, Finnin MS, Livingston DM, Pavletich NP (2002). Structure of the 53BP1 BRCT region bound to p53 and its comparison to the *BRCA1 *BRCT structure. Genes Dev.

[B35] Shiozaki EN, Gu L, Yan N, Shi Y (2004). Structure of the BRCT repeats of *BRCA1 *bound to a BACH1 phosphopeptide: implications for signaling. Mol Cell.

[B36] Williams RS, Lee MS, Hau DD, Glover JN (2004). Structural basis of phosphopeptide recognition by the BRCT domain of *BRCA1*. Nat Struct Mol Biol.

[B37] Varma AK, Brown RS, Birrane G, Ladias JA (2005). Structural basis for cell cycle checkpoint control by the *BRCA1 *-CtIP complex. Biochemistry.

[B38] Clapperton JA, Manke IA, Lowery DM, Ho T, Haire LF, Yaffe MB, Smerdon SJ (2004). Structure and mechanism of *BRCA1 *BRCT domain recognition of phosphorylated BACH1 with implications for cancer. Nat Struct Mol Biol.

[B39] Belogianni I, Apessos A, Mihalatos M, Razi E, Labropoulos S, Petounis A, Gaki V, Keramopoulos A, Pandis N, Kyriacou K, Hadjisavvas A, Kosmidis P, Yannoukakos D, Nasioulas G (2004). Characterization of a novel large deletion and single point mutations in the *BRCA1 *gene in a Greek cohort of families with suspected hereditary breast cancer. BMC Cancer.

[B40] Preisler-Adams S, Schönbuchner I, Fiebig B, Welling B, Dworniczak B, Weber BH (2006). Gross rearrangements in *BRCA1 *but not *BRCA2 *play a notable role in predisposition to breast and ovarian cancer in high-risk families of German origin. Cancer Genet Cytogenet.

[B41] Thompson D, Easton D, Breast Cancer Linkage Consortium (2002). Variation in *BRCA1 *cancer risks by mutation position. Cancer Epidemiol Biomarkers Prev.

[B42] Montagna M, Agata S, De Nicolo A, Menin C, Sordi G, Chieco-Bianchi L, D'Andrea E (2002). Identification of *BRCA1 *and *BRCA2 *by carriers by allele-specific gene expression (AGE) analysis. Int J Cancer.

[B43] Deng CX, Brodie SG (2000). Roles of *BRCA1 *and its interacting proteins. Bioessays.

[B44] Williams RS, Chasman DI, Duong Hau D, Hui B, Lau AY, Glover JNM (2003). Detection of protein folding defects caused by *BRCA1 *-BRCT truncation and missense mutations. J Biol Chem.

[B45] Williams RS, Green R, Glover JNM (2001). Crystal structure of the BRCT repeat region from the breast cancer-associated protein *BRCA1*. Nat Struct Biol.

[B46] Williams RS, Glover JNM (2003). Structural consequences of a cancer-causing *BRCA1 *-BRCT missense mutation. J Biol Chem.

[B47] Lahti-Domenici J, Rapakko K, Paakkonen K, Allinen M, Nevenlinna H, Kujala M, Huusko P, Wingwist R (2001). Exclusion of large deletions and other rearrangementsin *BRCA1 *and *BRCA2 *in Finnish breast and ovarian cancer families. Cancer Genet Cytogenet.

